# Seven Functional Polymorphisms in the *CETP* Gene and Myocardial Infarction Risk: A Meta-Analysis and Meta-Regression

**DOI:** 10.1371/journal.pone.0088118

**Published:** 2014-02-12

**Authors:** Qi Wang, Shao-Bo Zhou, Li-Jie Wang, Ming-Ming Lei, Yong Wang, Chi Miao, Yuan-Zhe Jin

**Affiliations:** Department of Cardiology, the Fourth Affiliated Hospital of China Medical University, Shenyang, P.R. China; University Medical Center Rotterdam, Netherlands

## Abstract

**Objective:**

This meta-analysis aims to evaluate the relationships between seven functional polymorphisms in the *CETP* gene and myocardial infarction (MI) risk.

**Method:**

The PubMed, CISCOM, CINAHL, Web of Science, Google Scholar, EBSCO, Cochrane Library, and CBM databases were searched for relevant articles published before March 1st, 2013 without any language restrictions. Meta-analysis was conducted using the STATA 12.0 software.

**Results:**

Nine case-control studies with a total 8,623 MI cases and 8,564 healthy subjects met the inclusion criteria. The results of our meta-analysis suggested that *CETP* rs708272 (C>T) polymorphism might be correlated with an increased risk of MI, especially among Caucasians. Furthermore, we observed that *CETP* rs1800775 (C>A) polymorphism might increase the risk of MI. Nevertheless, no similar findings were found for *CETP* rs5882 (A>G), rs2303790 (A>G), rs1800776 (C>A), rs12149545 (G>A), and rs4783961 (G>A) polymorphisms.

**Conclusion:**

The current meta-analysis suggests that *CETP* rs708272 (C>T) and rs1800775 (C>A) polymorphisms may contribute to MI susceptibility, especially among Caucasians. Thus, *CETP* rs708272 and rs1800775 polymorphisms may be promising and potential biomarkers for early diagnosis of MI.

## Introduction

Myocardial infarction (MI) remains the leading cause of death and disability worldwide, accounting for up to 40% of all deaths [1]. Due to high mortality and disability rates, MI is becoming a global epidemiological health concern [2]. Rupturing of coronary atherosclerotic plaque with consequent platelet aggregation and thrombus formation is the major cause of MI [3]–[5]. Many intrinsic and extrinsic risk factors for MI have been established, including dyslipidemia, hypertension, smoking, obesity, etc. [6], [7]. Atherogenic dyslipidemia is usually characterized by three lipid abnormalities: increases in plasma triglyceride, small low density cholesterol (LDL-C) and very low density lipoprotein cholesterol (VLDL-C) levels, and decreased high-density lipoprotein cholesterol (HDL-C) levels [8]–[10]. Although the exact cellular and molecular mechanisms leading to the development of MI remain unclear, it is believed that functionally relevant mutations in the dyslipidemia-related genes may contribute to increased susceptibility to MI [11].

Cholesteryl ester transfer protein (CETP) is a plasma protein that mediates the exchange of neutral lipids, including cholesteryl esters and triglycerides between plasma lipoproteins [12]. CETP plays a critical role in reverse cholesteryl transport of cholesteryl esters and triglycerides from HDL-C to LDL-C and VLDL-C [13], [14]. It is well established that HDL-C has a protective role against cardiovascular disease [15]. Plasma HDL particles play an important role in removing cellular cholesterol and delivering it to the liver for re-utilization [16]. Furthermore, it should be noted that levels of HDL-C is significantly negatively correlated with arterial stenosis whose occurrence is strongly associated with the phenomenon of plaque rupture [17]. Thus higher levels of HDL-C tend to have fewer problems with cardiovascular diseases such as MI, while those with low HDL-C cholesterol levels may easily suffer from MI [18], [19]. Variation in CETP activity could influence HDL-C levels and thus contribute to increased susceptibility to cardiovascular disease such as MI [20]. Genetic and epigenetic changes in the *CETP* gene may enhance plasma cholesteryl ester formation and lead to low HDL-C levels and thereby possibly explain the inter-individual differences in MI risk [21], [22].

Human *CETP* gene is located on chromosome 6q21 and consists of 16 exons and 15 introns, spanning approximately 25 kb [23], [24]. Some genetic variations in the *CETP* gene have been found in the *CETP* gene, such as rs708272 (C>T), rs1800775 (C>A), rs5882 (A>G), rs2303790 (A>G), rs1800776 (C>A), rs12149545 (G>A), and rs4783961 (G>A); among these, rs708272 (C>T) and rs1800775 (C>A) are the most common variants that have been widely investigated [25], [26]. Rs708272, a SNP in intron 1 (known as TaqIB), results from a C-to-T substitution at position 277 [27]; rs1800775 is a promoter SNP arising from a substitution of C-to-A at position 629 [28]. Many previous studies have demonstrated that *CETP* genetic polymorphisms might be a reliable predictor for the incidence of MI [29]–[32]. Nevertheless, contradictory results were also reported in many of the other studies [33]–[36]. Consequently, we performed the present meta-analysis to evaluate the relationships of seven functional polymorphisms in the *CETP* gene and the risk of MI.

## Materials and Methods

### Search strategy

The PubMed, CISCOM, CINAHL, Web of Science, Google Scholar, EBSCO, Cochrane Library, and CBM databases were searched for relevant articles published before March 1st, 2013 without any language restrictions. The following keywords and MeSH terms were used: (“SNP” or “mutation” or “genetic polymorphism” or “variation” or “polymorphism” or “single nucleotide polymorphism” or “variant”) and (“myocardial infarction” or “myocardial infarct” or “MI” or “AMI” or “heart attacks”) and (“cholesterol ester transfer protein” or “CETP” or “cholesteryl ester exchange protein” or “CE transport protein”). We also performed a manual search of the reference lists from the relevant articles to find other potential articles.

### Selection criteria

The included studies must meet all four of the following criteria: (1) the study design must be clinical cohort or case-control study that focused on the relationships of *CETP* genetic polymorphisms with the risk of MI; (2) all patients met the diagnostic criteria for MI; (3) the genotype frequencies of healthy controls should follow the Hardy-Weinberg equilibrium (HWE); (4) the study must provide sufficient information about the genotype frequencies. If the study could not meet the inclusion criteria, it would be excluded. The most recent or the largest sample size publication was included when the authors published several studies using the same subjects. The PRISMA checklist is available in Checklist S1.

### Data extraction

Relevant data were systematically extracted from all included studies by two observers by using a standardized form. The researchers collected the following data: language of publication, publication year of article, the first author's surname, geographical location, design of study, sample size, the source of the subjects, genotype frequencies, source of samples, genotyping method, evidence of HWE, etc.

### Quality assessment

Methodological quality was evaluated separately by two observers using the Newcastle-Ottawa Scale (NOS) criteria [37]. The NOS criteria included three aspects: (1) subject selection: 0∼4; (2) comparability of subject: 0∼2; (3) clinical outcome: 0∼3. NOS scores ranged from 0 to 9; and a score ≥7 indicate a good quality. The NOS criteria are available in File S1.

### Statistical analysis

The STATA version 12.0 (Stata Corp, College Station, TX, USA) software was used for meta-analysis. We calculated crude odds ratio (OR) with their 95% confidence interval (95%CI) to evaluate their relationships under 5 genetic models. Genotype frequencies of healthy controls were tested for the HWE using the *χ^2^* test. The statistical significance of pooled ORs was assessed by the Z test. The Cochran's *Q*-statistic and *I^2^* test were used to evaluate potential heterogeneity between studies [38]–[40]. If *Q*-test shows a *P*<0.05 or *I^2^* test exhibits >50% which indicates significant heterogeneity, the random-effect model was conducted, or else the fixed-effects model was used. We also performed subgroup and meta-regression analyses to investigate potential sources of heterogeneity. We conducted a sensitivity analysis to assess the influence of single studies on the overall ORs. Begger's funnel plots and Egger's linear regression test were used to investigate publication bias [41].

## Results

### Baseline characteristics of included studies

**Figure 1:**
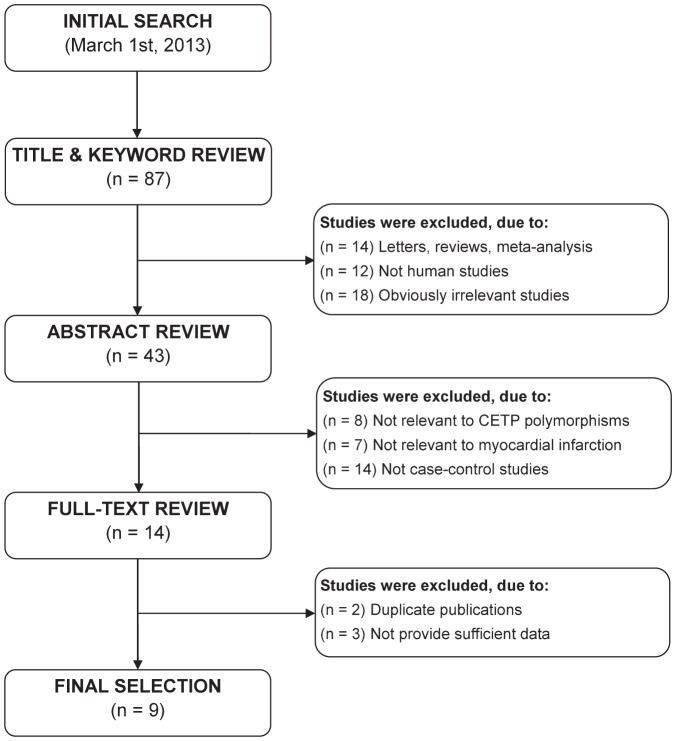
Flow chart shows study selection procedure.

Initially, the searched keywords identified 90 articles. We reviewed the titles and abstracts of all articles and excluded 44 articles; full texts were also reviewed and 34 articles were further excluded. Three other studies were excluded due to no sufficient data about seven common SNPs in the *CETP* gene [42]–[44]. Figure 1 shows the selection process of eligible articles. Finally, 9 case-control studies with a total 8,623 MI cases and 8,564 healthy subjects met our inclusion criteria for qualitative data analysis [29]–[36], [45]. Population-based controls were used in 6 studies, and hospital-based controls were used in 3 studies. Overall, seven studies were conducted among Caucasians and two studies among Asians. Polymerase chain reaction-restriction fragment length polymorphism (PCR-RELP) method was conducted in 7studies, and 2 studies used direct sequencing method. Seven common polymorphisms in the *CETP* gene were assessed, including rs708272 (C>T), rs1800775 (C>A), rs5882 (A>G), rs2303790 (A>G), rs1800776 (C>A), rs12149545 (G>A), and rs4783961 (G>A); and among these, rs708272 (C>T) and rs1800775 (C>A) were the most common SNPs. None of the studies deviated from the HWE (all *P*>0.05). NOS scores of all included studies were ≥5. We summarized the study characteristics and methodological quality in Table 1. The genotypic distributions of *CETP* genetic polymorphisms are shown in File S2.

**Table 1:** Main characteristics and methodological quality of all eligible studies.

First author [Ref]	Year	Country	Ethnicity	Number		Gender (M/F)		Age (years)		Source		Genotype method	SNP	NOS score
				Case	Control	Case	Control	Case	Control	Case	Control			
Li et al [36]	1999	China	Asian	102	102	81/21	82/20	59.0±7.5	57.0±7.0	HB	HB	PCR-RFLP	rs2303790 (A>G)	24
Zhang et al [35]	2005	China	Asian	50	94	35/15	52/42	65.0±10.0	54.0±18.0	HB	PB	PCR-RFLP	rs708272 (G>A)	26
Eiriksdottir et al [29]	2001	Iceland	Caucasian	388	749	388/0	749/0	71.0±0.01	76.0±0.01	PB	PB	PCR-RFLP	rs708272 (G>A)	30
													rs1800775 (C>A)	
Liu et al [33]	2002	USA	Caucasian	384	384	384/0	384/0	59.5±8.3	59.5±8.5	HB	HB	PCR-RFLP	rs708272 (G>A)	31
Andrikopoulos et al [34]	2004	Greek	Caucasian	1625	735	-	-	63.0±12.0	58.0±15.0	PB	PB	PCR-RFLP	rs5882 (A>G)	27
Keavney et al [30]	2004	UK	Caucasian	4442	3273	2892/1550	1453/1820	50.5±0.12	46.2±0.14	HB	HB	PCR-RFLP	rs708272 (G>A)	35
Tobin et al [31]	2004	UK	Caucasian	547	505	372/175	313/192	61.9±9.2	58.6±10.7	HB	PB	PCR-RFLP	rs1800776 (C>A)	33
													rs1800775 (C>A)	
													rs5882 (A>G)	
Zee et al [45]	2006	USA	Caucasian	523	2092	523/0	2092/0	58.3±0.4	58.4±0.2	PB	PB	Direct sequencing	rs1800775 (C>A)	33
Meiner et al [32]	2008	USA	Caucasian	561	629	314/237	294/335	44.0±5.2	42.2±5.3	PB	PB	Direct sequencing	rs12149545 (G>A)	34
													rs4783961 (G/A)	
													rs1800775 (C>A)	
													rs708272 (G>A)	
													rs5882 (A>G)	

M = male, F = female, PB = population-based, HB = hospital-based, PCR = polymerase chain reaction, RFLP = restriction fragment length polymorphism, SNP = single nucleotide polymorphism, NOS = Newcastle-Ottawa quality assessment scale.

### Quantitative data synthesis

**Figure 2:**
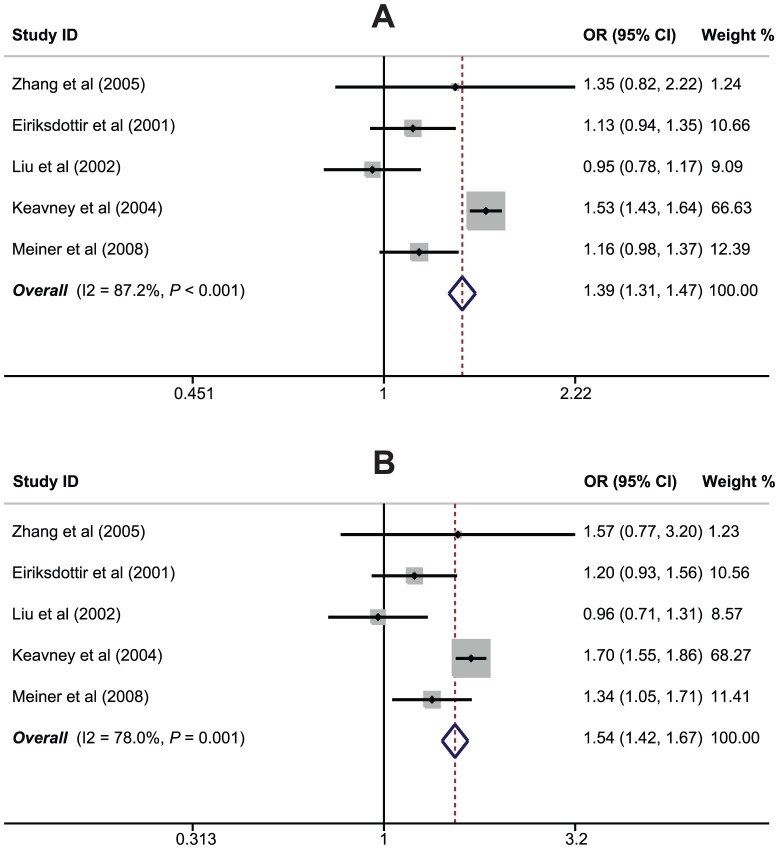
Forest plot of the relationships between *CETP* rs708272 (C>T) polymorphism and myocardial infarction risk under the allele and dominant models.

**Figure 3:**
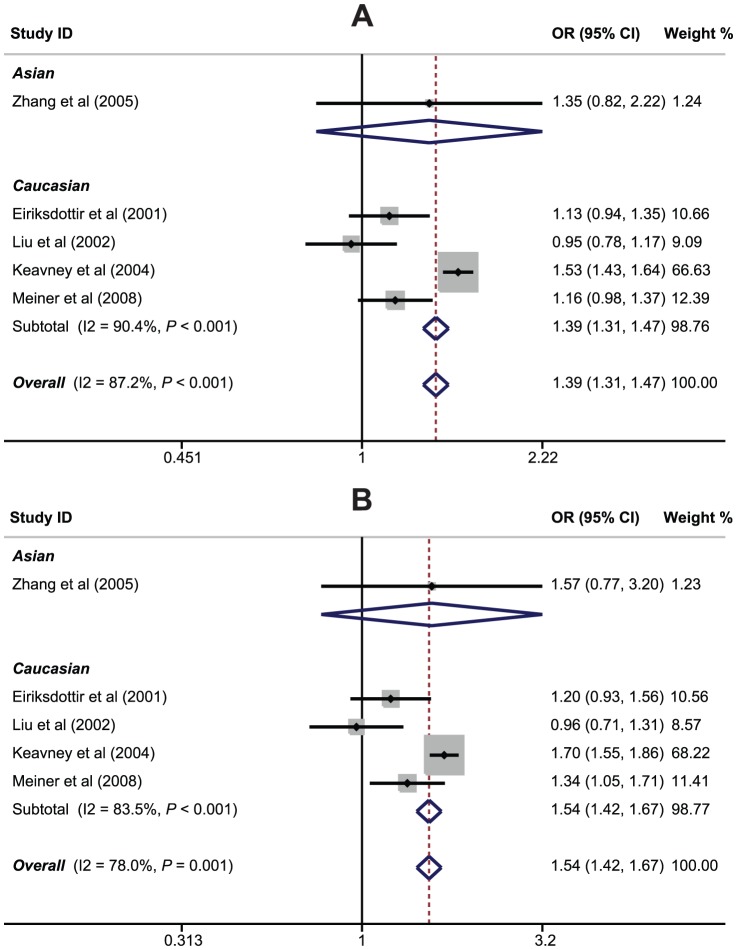
Subgroup analysis by ethnicity for the relationships between *CETP* rs708272 (C>T) polymorphism and myocardial infarction risk under the allele and dominant models.

The relationships of *CETP* rs708272 (C>T) polymorphism with the risk of MI were reported in 5 studies. The heterogeneity obviously existed (*P*<0.05), so the random effects model was conducted. Our meta-analysis results revealed that *CETP* rs708272 polymorphism may increase the risk of MI (T allele vs. C allele: OR = 1.39, 95%CI: 1.31–1.47, *P*<0.001; CT+TT vs. CC: OR = 1.54, 95%CI: 1.42–1.67, *P*<0.001; TT vs. CC+CT: OR = 1.52, 95%CI: 1.37–1.70, *P*<0.001; TT vs. CC: OR = 1.87, 95%CI: 1.66–2.11, *P*<0.001; TT vs. CT: OR = 1.29, 95%CI: 1.15–1.45, *P*<0.001) (Figure 2). Among different ethnic subgroups, the results revealed positive correlations between *CETP* rs708272 (C>T) polymorphism and an increased risk of MI among Caucasians (Figure 3), but not among Asians (all *P*>0.05). The results of subgroup analyses also suggested that *CETP* rs708272 (C>T) polymorphism was associated with increased risk of MI in the UK, population-based, hospital-based, PCR-RFLP and direct sequencing subgroups (as shown in Table 2). However, *CETP* rs708272 (C>T) polymorphism showed no association with MI susceptibility in studies conducted in China, Iceland and USA.

**Table 2:** Meta-analysis of the associations between *CETP* rs708272 (G>A) and rs1800775 (C>A) polymorphisms and endometrial cancer risk.

Subgroups	M allele vs. W allele (allele model)			WM+MM vs. WW (dominant model)			MM vs. WW+WM (recessive model)			MM vs. WW (homozygous model)			MM vs. WM (heterozygous model)		
	OR	[95%CI]	*P*	OR	[95%CI]	*P*	OR	[95%CI]	*P*	OR	[95%CI]	*P*	OR	[95%CI]	*P*
**rs708272 (G>A)**															
Overall	1.39	[1.31, 1.47]	<0.001	1.54	[1.42, 1.67]	<0.001	1.52	[1.37, 1.70]	<0.001	1.87	[1.66, 2.11]	<0.001	1.29	[1.15, 1.45]	<0.001
*Ethnicity*															
Caucasians	1.39	[1.31, 1.47]	<0.001	1.54	[1.42, 1.67]	<0.001	1.53	[1.37, 1.70]	<0.001	1.87	[1.66, 2.11]	<0.001	1.30	[1.15, 1.46]	<0.001
Asians	1.35	[0.82, 2.22]	0.243	1.57	[0.77, 3.20]	0.216	1.30	[0.49, 3.42]	0.594	1.65	[0.57, 4.74]	0.355	1.07	[0.38, 2.97]	0.902
*Country*															
China	1.35	[0.82, 2.22]	0.243	1.57	[0.77, 3.20]	0.216	1.30	[0.49, 3.42]	0.594	1.65	[0.57, 4.74]	0.355	1.07	[0.38, 2.97]	0.902
Iceland	1.13	[0.94, 1.35]	0.185	1.20	[0.93, 1.56]	0.162	1.13	[0.80, 1.59]	0.497	1.25	[0.85, 1.83]	0.257	1.05	[0.73, 1.51]	0.803
USA	1.07	[0.94, 1.22]	0.290	1.17	[0.98, 1.42]	0.088	0.98	[0.77, 1.24]	0.852	1.09	[0.84, 1.42]	0.516	0.90	[0.70, 1.16]	0.428
UK	1.53	[1.43, 1.64]	<0.001	1.70	[1.55, 1.86]	<0.001	1.83	[1.60, 2.10]	<0.001	2.32	[2.01, 2.68]	<0.001	1.50	[1.30, 1.73]	<0.001
*Source of controls*															
Population-based	1.16	[1.03, 1.30]	0.017	1.29	[1.09, 1.53]	0.004	1.09	[0.87, 1.36]	0.471	1.27	[0.99, 1.62]	0.062	0.97	[0.77, 1.23]	0.809
Hospital-based	1.46	[1.37, 1.56]	<0.001	1.62	[1.48, 1.77]	<0.001	1.69	[1.49, 1.92]	<0.001	2.10	[1.83, 2.40]	<0.001	1.41	[1.24, 1.62]	<0.001
*Genotype methods*															
PCR-RFLP	1.42	[1.34, 1.51]	<0.001	1.57	[1.44, 1.70]	<0.001	1.61	[1.43, 1.81]	<0.001	1.98	[1.74, 2.24]	<0.001	1.36	[1.20, 1.54]	<0.001
DNA sequencing	1.16	[0.98, 1.37]	0.081	1.34	[1.05, 1.70]	0.018	1.04	[0.76, 1.41]	0.821	1.25	[0.89, 1.75]	0.205	0.91	[0.66, 1.25]	0.554
**rs1800775 (C>A)**															
Overall	1.13	[1.05, 1.22]	0.002	1.34	[1.18, 1.53]	<0.001	1.03	[0.90, 1.17]	0.713	1.27	[1.08, 1.49]	0.004	0.92	[0.80, 1.06]	0.239
*Country*															
Iceland	1.31	[1.10, 1.56]	0.003	1.74	[1.30, 2.33]	<0.001	1.18	[0.88, 1.58]	0.263	1.71	[1.20, 2.45]	0.003	0.98	[0.72, 1.33]	0.883
USA	1.21	[1.09, 1.34]	<0.001	0.89	[0.67, 1.18]	<0.001	1.15	[0.97, 1.36]	0.119	1.45	[1.18, 1.79]	0.001	1.02	[0.85, 1.22]	0.853
UK	0.83	[0.70, 0.98]	0.028	1.43	[1.21, 1.69]	0.415	0.66	[0.50, 0.88]	0.005	0.67	[0.47, 0.95]	0.024	0.66	[0.49, 0.89]	0.007
*Genotype methods*															
PCR-RFLP	1.03	[0.92, 1.17]	0.592	1.24	[1.01, 1.51]	0.038	0.88	[0.72, 1.08]	0.215	1.05	[0.82, 1.35]	0.678	0.80	[0.65, 0.99]	0.043
DNA sequencing	1.21	[1.09, 1.34]	<0.001	1.43	[1.21, 1.69]	<0.001	1.15	[0.97, 1.36]	0.119	1.45	[1.18, 1.79]	0.001	1.02	[0.85, 1.22]	0.853

OR = odds ratios, 95%CI = 95% confidence interval, W = wild allele, M = mutant allele, WW = wild homozygote, WM = heterozygote, MM = mutant homozygote, PCR = polymerase chain reaction, RFLP = restriction fragment length polymorphism.

**Figure 4:**
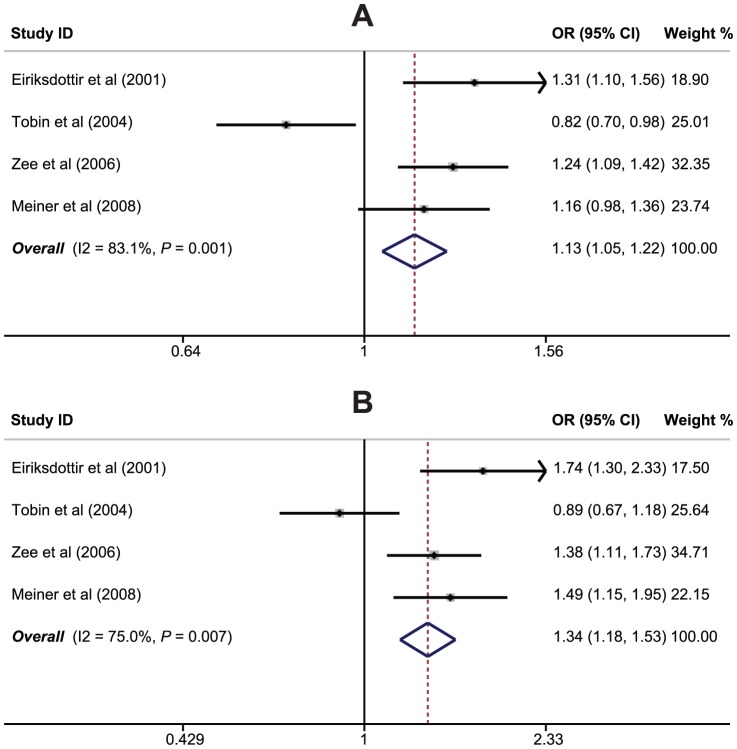
Forest plot of the relationships between *CETP* rs1800775 (C>A) polymorphism and myocardial infarction risk under the allele and dominant models.

There were 4 studies that referred to the relationships of *CETP* rs1800775 (C>A) polymorphism with MI risk. Since heterogeneity was significantly observed (*P*<0.05), the random effects model was used. Meta-analysis of these studies indicated positive correlations of *CETP* rs1800775 (C>A) polymorphism with an increased risk of MI (A allele vs. C allele: OR = 1.13, 95%CI: 1.05–1.22, *P* = 0.002; CA+AA vs. CC: OR = 1.34, 95%CI: 1.18–1.53, *P*<0.001; AA vs. CC: OR = 1.27, 95%CI: 1.08–1.49, *P* = 0.004) (Figure 4). We also conducted subgroup analyses by country and genotype; the results indicated that *CETP* rs1800775 (C>A) polymorphism might increase susceptibility to MI in most subgroups (as shown in Table 2).

The relationships of rs5882 (A>G), rs2303790 (A>G), rs1800776 (C>A), rs12149545 (G>A), and rs4783961 (G>A) polymorphisms with MI risk were also evaluated. Nevertheless, no similar associations were found for *CETP* rs5882 (A>G), rs2303790 (A>G), rs1800776 (C>A), rs12149545 (G>A), and rs4783961 (G>A) polymorphisms (all *P*>0.05) (as shown in Table 3).

**Table 3:** Meta-analysis of the associations between five common polymorphisms in *CETP* gene and MI risk.

SNP ID	M allele vs. W allele (allele model)			WM+MM vs. WW (dominant model)			MM vs. WW+WM (recessive model)			MM vs. WW (homozygous model)			MM vs. WM (heterozygous model)		
	OR	[95%CI]	*P*	OR	[95%CI]	*P*	OR	[95%CI]	*P*	OR	[95%CI]	*P*	OR	[95%CI]	*P*
rs2303790 (A>G)	1.52	[0.42, 5.45]	0.525	1.53	[0.42, 5.60]	0.519	-	-	-	-	-	-	-	-	-
rs5882 (A>G)	0.96	[0.88, 1.05]	0.343	0.95	[0.84, 1.07]	0.420	0.93	[0.78, 1.12]	0.461	0.91	[0.75, 1.11]	0.365	0.95	[0.78, 1.15]	0.606
rs1800776 (C>A)	1.10	[0.80, 1.50]	0.564	1.10	[0.79, 1.53]	0.593	1.85	[0.17, 20.46]	0.616	1.87	[0.17, 20.73]	0.609	1.73	[0.15, 19.43]	0.658
rs12149545 (G>A)	0.93	[0.78, 1.12]	0.448	0.92	[0.74, 1.16]	0.493	0.87	[0.55, 1.40]	0.573	0.85	[0.52, 1.37]	0.501	0.91	[0.56, 1.48]	0.692
rs4783961 (G>A)	0.89	[0.76, 1.04]	0.146	0.83	[0.64, 1.07]	0.154	0.89	[0.69, 1.15]	0.365	0.80	[0.58, 1.09]	0.156	0.94	[0.72, 1.24]	0.677

OR = odds ratios, 95%CI = 95% confidence interval, W = wild allele, M = mutant allele, WW = wild homozygote, WM = heterozygote, MM = mutant homozygote, SNP = single nucleotide polymorphism.

**Figure 5:**
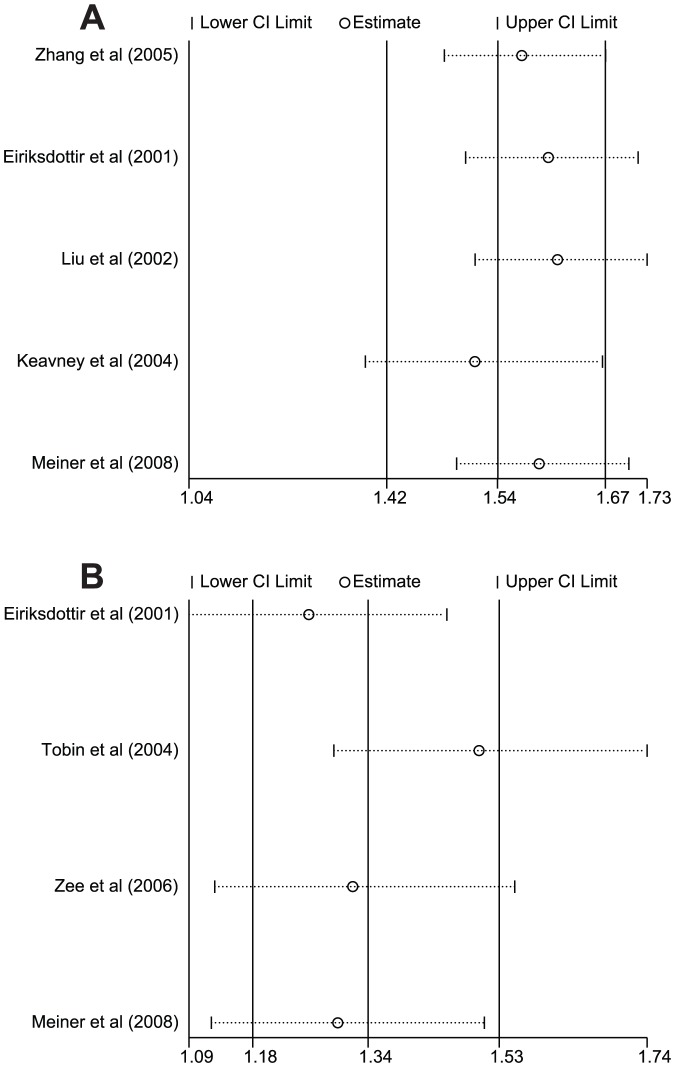
Sensitivity analysis of the relationships of *CETP* rs708272 (C>T) and rs1800775 (C>A) polymorphisms with myocardial infarction risk.

**Figure 6:**
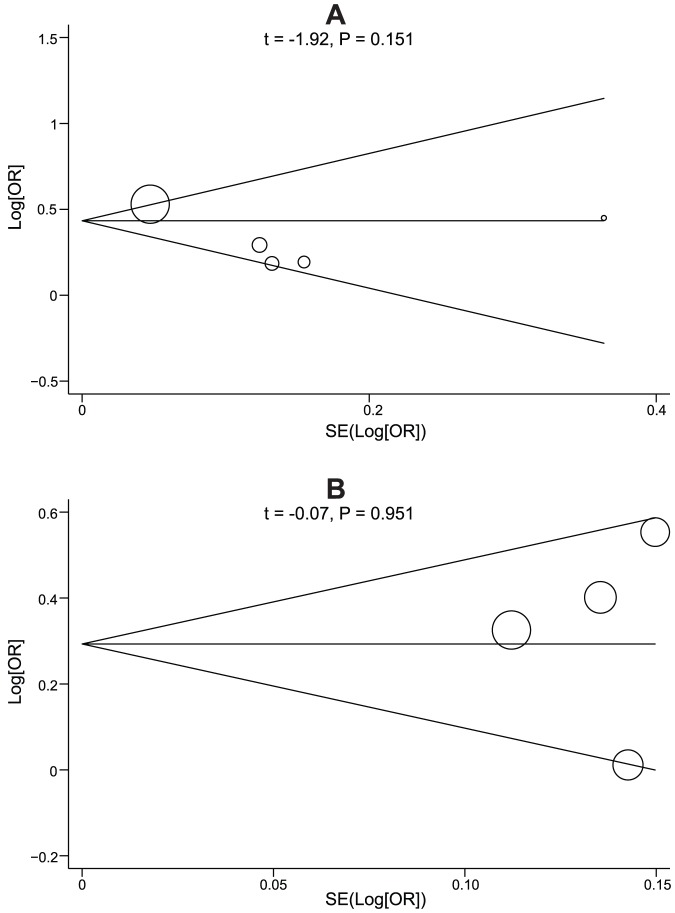
Begger's funnel plots of the relationships of *CETP* rs708272 (C>T) and rs1800775 (C>A) polymorphisms with myocardial infarction risk. Each point represents a separate study for the indicated association. Log[OR], natural logarithm of OR. Horizontal line, mean magnitude of the effect.

Meta-regression analyses were conducted for rs708272 (C>T) and rs1800775 (C>A) polymorphisms. The results confirmed that ethnicity might be a main source of heterogeneity (as shown in Table 4). The results of sensitivity analysis indicated that the overall pooled ORs could not be affected by single study (Figure 5). No evidence for asymmetry was observed in the Begger's funnel plots (Figure 6). Egger's test also failed to reveal any evidence of publication bias (rs708272: *t* = −1.92, *P* = 0.151; rs1800775: *t* = −0.07, *P* = 0.951).

**Table 4:** Univariate and multivariate meta-regression analyses of potential source of heterogeneity.

Heterogeneity factors	rs708272 (C>T)				rs1800775 (C>A)			
	β [95%CI]	SE	z	*P*	β [95%CI]	SE	z	*P*
*Publication year*								
Univariate	0.032 [−0.058, 0.124]	0.046	0.71	0.478	−0.009 [−0.141, 0.122]	0.067	−0.14	0.889
Multivariate	0.014 [−0.113, 0.143]	0.065	0.23	0.821	−0.003 [−0.131, 0.126]	0.066	−0.04	0.966
*Ethnicity*								
Univariate	0.182 [−0.700, 1.065]	0.507	3.24	0.001	−0.542 [−0.877, 0.209]	0.170	−3.18	0.001
Multivariate	0.500 [−0.912, 1.911]	0.441	2.69	0.007	−0.559 [−0.993, 0.125]	0.222	−2.52	0.012
*Country*								
Univariate	0.185 [0.073, 0.297]	0.450	0.41	0.685	−0.039 [−0.446, 0.368]	0.208	−0.19	0.850
Multivariate	0.160 [−0.309, 0.628]	0.239	0.67	0.504	−0.150 [−2.237, 1.935]	1.064	−0.14	0.887
*Source of controls*								
Univariate	−0.021 [−0.506, 0.465]	0.248	−0.08	0.934	0.355 [−0.114, 0.824]	0.239	1.48	0.138
Multivariate	0.192 [−0.195, 0.579]	0.197	0.97	0.330	0.362 [−0.296, 1.021]	0.336	1.08	0.281
*Genotype methods*								
Univariate	−0.013 [−0.600, 0.574]	0.300	−0.04	0.966	−0.147 [−0.790, 0.496]	0.328	−0.45	0.654
Multivariate	1.189 [0.324, 2.054]	0.720	0.69	0.488	−0.768 [−2.000, 0.463]	0.628	−1.22	0.221

SE = standard error, 95%CI = 95% confidence interval.

## Discussion

CETP, a hydrophobic glycoprotein secreted mainly by the liver, catalyzes the transfer of cholesteryl esters from HDL to other lipoproteins and influences plasma HDL-C levels [46], [47]. Previous studies have demonstrated a protective effect of HDL-C against cardiovascular disease by inhibiting lipoprotein oxidation [8], [48], [49]. High plasma levels of CETP are correlated with low HDL-C levels, and it has been implicated as a strong risk factor for cardiovascular disease, including MI [50]. Although MI is one of the most common heritable cardiovascular diseases, the fundamental molecular pathways remain undefined [51], [52]. Thus, it was speculated that *CETP* genetic variations may be involved in the development of MI [45], [53]. The *CETP* gene has been mapped to locus 16q21 encoding cholesteryl ester transfer protein [23]. Common polymorphisms of *CETP* gene may result in the over-expression of this protein and a subsequent decrease of HDL-C levels, thus contributing to the incidence of MI [21]. Indeed, several studies have demonstrated positive correlations of *CETP* genetic polymorphisms with an increased risk of MI [29]–[32], but the controversy still persists.

In the present meta-analysis, our findings revealed that *CETP* rs708272 (C>T) polymorphism might increase the risk of MI, especially among Caucasians, while similar results were not observed among Asians. There also existed positive correlations of *CETP* rs1800775 (C>A) polymorphism with an increased risk of MI among Caucasians. Although ethnic differences in to the risk of MI are well known, potential molecular mechanism is not fully understood. One possible reason for ethnic difference might be that *CETP* gene mutations might affect cholesteryl ester synthesis and result in low HDL-C levels, thereby possibly explaining interindividual differences in the incidence of MI [21]. Another likely explanation for this difference could be that large differences in common SNPs that influence the risk of MI are mostly due to genetic drift and natural selection [54]. The results of subgroup analyses demonstrated positive correlations of *CETP* rs708272 (C>T) polymorphism with an increased risk of MI in the UK, population-based, hospital-based, PCR-RFLP and direct sequencing subgroups, indicating that country, source of controls and genotype method may be the potential sources of heterogeneity. However, our meta-regression analyses indicated that only ethnicity was the major source of heterogeneity. These disparate results may be due to small sample size resulting in substantial errors from estimation. Nevertheless, we observed no associations between the other 5 common polymorphisms in the *CETP* gene and MI risk. In short, the results of our meta-analysis were consistent with previous studies that *CETP* genetic polymorphisms may be closely linked to the risk of MI, suggesting that *CETP* genetic polymorphism could be useful and promising biomarkers for early diagnosis of MI.

The current meta-analysis also had many limitations that should be acknowledged. First, our results had lacked sufficient statistical power to assess the correlations between *CETP* genetic polymorphisms and MI risk. Secondly, meta-analysis is a retrospective study that may lead to subject selection bias, and thereby affecting the reliability of our results [55]. Thirdly, our meta-analysis failed to obtain original data from the included studies, which may limit further evaluation of potential role of *CETP* genetic polymorphisms in the development of MI. Although our study has many limitations, this is the first meta-analysis focusing on the relationships between *CETP* genetic polymorphisms and the risk of MI. Furthermore, we performed a highly sensitive literature search strategy for electronic databases. A manual search of the reference lists from the relevant articles was also conducted to find other potential articles. The selection process of eligible articles was based on strict inclusion and exclusion criteria. Importantly, rigorous statistical analysis of SNP data provided a basis for pooling of information from individual studies.

In conclusion, our findings provide empirical evidence that *CETP* rs708272 (C>T) and rs1800775 (C>A) polymorphisms may contribute to MI susceptibility, especially among Caucasians. Thus, *CETP* rs708272 and rs1800775 polymorphisms may be promising and potential biomarkers for early diagnosis of MI. However, due to the limitations mentioned above, more researches with larger sample size are needed to provide a more representative statistical analysis precisely.

## Supporting Information

Checklist S1 The PRISMA Checklist.
(DOC)

File S1 The Newcastle-Ottawa quality assessment scale.
(DOC)

File S2 The genotypic distributions of *CETP* genetic polymorphisms in the case and control groups.
(XLS)
